# Serum trace elements in obese Egyptian children: a case–control study

**DOI:** 10.1186/1824-7288-40-20

**Published:** 2014-02-20

**Authors:** Seham FA Azab, Safaa H Saleh, Wafaa F Elsaeed, Mona A Elshafie, Laila M Sherief, Asmaa MH Esh

**Affiliations:** 1 Faculty of Medicine, Zagazig University, 18 Omar Bin Elkhattab St, Al Qawmia, Zagazig City, Sharkia Governorate, Egypt; 2 Faculty of Medicine, Zagazig University, Zagazig, Egypt

**Keywords:** Trace elements, Obesity, Children

## Abstract

**Background:**

To date, only a few studies on child obesity concerned Trace Elements (TE). TE is involved in the pathogenesis of obesity and obesity related diseases. We tried to assess trace elements status [zinc (Zn), copper (Cu), selenium (Se), iron (Fe), and chromium (Cr)] in obese Egyptian children and their relationships with serum leptin and metabolic risk factors of obesity.

**Methods:**

This was a case–control study performed with 80 obese children (BMI ≥ 95^th^centile for age and gender) and 80 healthy non-obese children with comparable age and gender as the control group. For all subjects, serum Zn, Cu, Se, Fe, ferritin and Cr as well as biochemical parameters including lipid profile, serum glucose and homeostasis model assessment of insulin resistance (HOMA-IR) were assessed. Levels of serum leptin were measured by (enzyme-linked immunosorbent assay [ELISA] method), and serum insulin was measured by an electrochemiluminesce immunoassay.

**Results:**

Compared to the control group, serum Zn, Se, and Fe levels were significantly lower (*all P* < 0.01) and serum Cu level was significantly higher (*P* < 0.01) in the obese children. Meanwhile, no significant differences were observed in serum ferritin or Cr levels (*P* > 0.05). A significant negative correlation was found between serum leptin and zinc levels in the obese children (*r* = −0.746; *P* < 0.01). Further, serum Zn showed significant negative correlations with total cholesterol TC levels (*P* < 0.05) and were positively correlated with high density lipoprotein- cholesterol HDL-C levels (*P* < 0.01) in the obese children. In addition, serum Se levels showed significant positive correlations with HOMA-IR values in the obese children (*P* < 0.01).

**Conclusion:**

The obese children may be at a greater risk of developing imbalance (mainly deficiency) of trace elements which may be playing an important role in the pathogenesis of obesity and related metabolic risk factors.

## Background

Childhood obesity has been called “one of the most serious public health challenges of the 21st century” [1]. According to the report of International Obesity Task Force (IOTF), in the year 2000 about 10% (a total of 155 million) of the young people aged 5–17 years globally were overweight; among whom 2-3% (30–45 million) were obese, a further 22 million younger children are also affected according to previous IOTF global estimates based on WHO data for under-fives [2,3]. Developing countries are facing the double burden of nutritional transition, i.e. malnutrition and micronutrient deficiencies as well as a rapidly growing epidemic of childhood obesity [4]. Genetic factors, environmental factors, lifestyle preferences, and cultural factors seem to play major role in the rising prevalence of obesity worldwide [5,6]. Leptin, a 16 kDa neurohormone predominantly synthesized and released into blood by adipocytes and serves as a signal for the brain of the body’s energy store. Leptin controls food intake through its receptors in the hypothalamus by inhibiting the release of NPY which has an augmentative effect on food intake. By reducing food intake and increasing thermogenesis, leptin is a key hormone in the regulation of body weight and nutrition [7]. Interest in trace elements has been steadily increasing over the last 25 years. Trace elements are essential nutrients with regulatory, immunologic, and antioxidant functions resulting from their action as essential components or cofactors of enzymes throughout metabolism [8]. To date, only a few studies on child obesity concerned Trace Elements (TE). TE are involved in the production of or in the protection against inflammation and peroxidation which are key factors in the development of the metabolic complications of obesity, arterial hypertension, dyslipidemia and insulin resistance or diabetes [9]. Therefore, it is important to know and measure trace elements status in children because the alterations in the content may play an important role in the pathogenesis of obesity and related metabolic risk factors. In this study, we aimed to estimate serum trace elements status [zinc (Zn), copper (Cu), selenium (Se), iron (Fe), and chromium (Cr)] in obese Egyptian children in comparison with normal weight controls. Further, correlations between serum trace elements levels and body mass index (BMI), serum leptin, lipid profile, fasting glucose, insulin and homeostasis model assessment of insulin resistance (HOMA-IR) were also determined in these children.

## Methods

This was a case–control study performed in Zagazig University Children Hospital Outpatient Clinics from May 2011 to April 2013. Subjects were children attending the specialized obesity outpatient clinic of Zagazig University Hospital. They were all apparently healthy with no obvious endocrine disease. Our study included 80 obese subjects with BMI ≥ 95^th^ centile for age and sex (36 girls and 44 boys). The age of the obese children ranged from 5.5 to 10 years (mean, 7.8 years). Eighty non obese children of comparable age and sex who were found healthy after the evaluation in pediatric clinics were included as controls.

### Exclusion criteria

Children with syndromal obesity, endocrine disorders, any physical disability, history of chronic medication use, use of mineral and/or vitamin supplements, history of any chronic diseases and/or chronic medication use or children under special diets were not included in the study.

A detailed medical and family history was obtained from all subjects. At enrollment, obese and control subjects underwent physical examination including weight, standing height, BMI, and blood pressure measurements.

### Anthropometrics

Height was measured without shoes using a Harpenden stadiometer (Harpenden, Holtain Ltd., UK) to the nearest 0.1 cm. Weight was measured to the nearest 0.1 kg on a standard beam scale with the subject dressed only in light underwear and without shoes. The waist circumference was measured at its smallest point between the iliac crest and rib cage, and the hip circumference was measured at its largest width over the greater trochanters. All the measurements were repeated twice. The weight status was recorded as the BMI, calculated as follows: BMI = weight (kg)/height(m)^2^. Because the BMI varies according to age, we standardized the value for age and sex by converting to a “z score” [10] and expressed the value as the BMI-SDS, which was calculated as follows: BMI-SDS = [individual measurement − population mean]/population SD. Overweight was defined as BMI between 1 and 1.99 SD and obesity above 2 SD. The distribution of fat mass was expressed by the waist-to-hip ratio (waist circumference/hip circumference).

Pubertal stage was assessed according to Tanner criteria. All our subjects (obese and control groups) were in the pre-pubertal stage (boys with pubic hair and gonadal stage I, girls with pubic hair and breast stage I).

### Biochemical analysis

On the first visit after enrollment, blood sample was drawn after an overnight fast from both groups. Serum lipids, glucose and insulin levels were measured immediately. Blood samples were drawn using metal-free and stainless steel needles into appropriately coated tubes (Becton Dickinson Laboratories, Franklin Lakes, NJ,USA) for measurement of serum levels of zinc, copper, selenium, iron, and chromium in obese and control children. The tubes were centrifuged at 2,000 × g for 10 minutes and the samples were tested for trace elements using Inductively Coupled Plasma mass-spectrometry (ICP-MS) (Perkin Emler Optima 4300, DV, USA) [11]. Serum Ferritin was analyzed by an immuno-assay (Elecsys, Boehringer Manneim, France).

### Serum leptin and insulin measurement

Serum Leptin was detected with commercially available test kits were used to measure leptin (enzyme-linked immunosorbent assay [ELISA] method, DRG International, Mountainside, NJ, USA), that has a range of 0.25.120 ng/mL. The DRG Leptin ELISA is an enzyme immunoassay for the measurement of leptin in serum and plasma. In the DRG Leptin ELISA the following values are observed: males 3.84 ± 1.79 ng/mL, females 7.36 ± 3.73 ng/Ml [12]. Fasting serum insulin was measured by electrochemiluminescence immunoassay using Cobas e 411 immunoassay analyzer (Roch Diagnostics, GmbH, German). Fasting glucose, total cholesterol (TC) and triglycerides (TGs) concentrations were determined in plasma with commercial kits (Elitech, Sees, France) using a clinical chemical analyzer (Bayer RA-50, Bayer Diagnostics, Dulin, Ireland). Both, high density lipoprotein- cholesterol (HDL-C) and low density lipoprotein- cholesterol (LDL-C) were measured by spectrophotometry (Genesis 20 ThermoSpectronic, Thermo Electron Corp, Madison, WI) with commercially available kits (Cholesterol HDL, Elitech, Sees, France; Cholesterol LDL, Spinreact, Sant Esteve de Bas, Spain). High total cholesterol with concentrations >200 mg/dL, high LDL with concentrations > 100 mg/dL, high triglycerides with concentrations >150 mg/dL, and low HDL with concentrations <50 mg/dL [13].

The homeostasis model assessment of insulin resistance was calculated with the following formula (HOMA ‒ IR) = [fasting insulin (uU/ml) × fasting glucose (mmol/L)]/22.5 [14].

### Ethics

Informed parental consent was obtained to be eligible for enrollment into the study. The study was done according to the rules of the Local Ethics Committee of Faculty of Medicine, Zagazig University, Egypt.

### Statistical methods

SPSS version 19 and EPI-info Version 6.04 were used for data analysis. The data are expressed as the mean ± SD or median (min-max) where appropriate. Test selection was based on evaluating the variables for normal distribution using the *Shapiro-Wilk test*. If the variables had a normal distribution, *Student’s t-test* was used. If the variable did not have a normal distribution, the analysis was done using the *Mann–Whitney U test*. Categorical data were evaluated by *Pearson’s chi-square test*. Statistical correlations were calculated by *Pearson’s correlation test. P* < 0.05 was considered significant.

## Results

Our study included 80 obese subjects (36 girls and 44 boys, mean age 7.8 ± 2.3 years, mean body mass index [BMI] 28.8 ± 2.6 kg/m^2^, mean BMI- *z score* 3.7 ± 0.6, all pre-pubertal stage) and 80 non-obese control subjects (40 girls and 40 boys, mean age 8.1 ± 1.9 years, mean BMI 16.2 ± 2.4 kg/m^2^, mean BMI- *z score* 0.32 ±0.4, all pre-pubertal stage) whose clinical characteristics are listed in Table 1. The obesity and control groups showed no significant differences in terms of age or gender (*P* > 0.05). Compared to the controls, the obese children demonstrated significant differences in a number of clinical risk factors including body weight, BMI, BMI- *z score*, waist circumference, hip circumference, and waist/hip ratio (all p < 0.01; Table 1).

**Table 1:** Baseline clinical data of the obese children and non-obese controls

	**Obese children (n = 80)**	**Controls (n = 80)**	***P***
*Age (years)*	7.8 ± 2.3	8.1 ± 1.9	>0.05^a^
*Male/female*	44(55%)/36(45%)	40(50%)/40(50%)	>0.05^b^
*BMI (kg/m*^*2*^*)*	28.8 ± 2.6	16.2 ± 2.4	<0.01^a^
*BMI z score*	3.7 ± 0.6	0.32 ±0.4	<0.01^a^
Waist circumference *(cm)*	87.5 ± 8.7	59.6 ± 7.6	<0.01^a^
Hip circumference *(cm)*	97.3 ± 5.4	73.5 ± 4.8	<0.01^a^
Waist/hip ratio	0.89 ± 0.06	0.81 ± 0.05	<0.01^a^

BMI, body mass index; Values are mean ± standard deviations. ^a^Student’s *t*-test. ^b^Chi-square test. *P* value < 0.05 indicates a significant difference.

Serum Cu levels, triglycerides, total cholesterol, LDL-C, fasting blood glucose, fasting blood insulin, and HOMA-IR in obese children were significantly higher (all *P* < 0.01), whereas serum Zn, Se, Fe levels and HDL-cholesterol, were significantly lower than those of healthy controls (all *P* < 0.01). However, no significant differences were observed in serum ferritin or Cr levels between both groups (*P* > 0.05; Table 2). Serum leptin was significantly higher in the obese children compared to the control group (25.6 ± 3.2 vs 9.3 ± 1.5 ng/mL; *P* < 0.01) (Table 2).

**Table 2:** Baseline laboratory data of the obese children and non-obese controls

	**Obese children (n = 80)**	**Controls (n = 80)**	***P***
*Serum zinc (ug/dl)*	57 ± 14	75 ± 17	<0.01^a^
*Serum copper (ug/dl)*	123.7 ± 28	96.4 ± 23	<0.01^a^
*Serum selenium (ug/L)*	63.6 ± 15	78.3 ± 18	<0.01^a^
*Serum iron (ug/dl)*	46.8 ± 11	63.5 ± 13	<0.01^a^
*Serum ferritin (ng/ml)*	68.5 ± 11.2	71.6 ± 10.6	>0.05^a^
*Serum chromium (ng/ml)*	0.2 ± 0.08	0.18 ± 0.05	>0.05^a^
*Triglycerides (mg/dl)*	(294–68)173	(155–45)83	<0.01^c^
*Total cholesterol (mg/dl)*	(312–105)183	(178–67)98	<0.01^c^
*LDL- cholesterol (mg/dl)*	(228–43)97	(105–39)73	<0.01^c^
*HDL- cholesterol (mg/dl)*	(72–17)33	(91–40)56	<0.01^c^
*Fasting glucose (mg/dl)*	93.5 ± 8.3	81.5 ± 7.7	<0.01^a^
*Fasting insulin (uU/ml)*	13.8 ± 1.2	6.3 ± 0.8	<0.01^a^
*HOMA-IR*	3.1 ± 0.8	1.3 ± 0.5	<0.01^a^
*Serum leptin (ng/mL)*	25.6 ± 3.2	9.3 ± 1.5	<0.01^a^

LDL, low density lipoprotein; HDL, High density lipoprotein; HOMA-IR, homeostasis model assessment of insulin resistance. Values are mean ± standard deviations or median (minimum- maximum). *P* value < 0.05 indicates a significant difference. ^a^Student’s *t*-test. ^c^Mann–Whitney *u* test.

**Figure 1:**
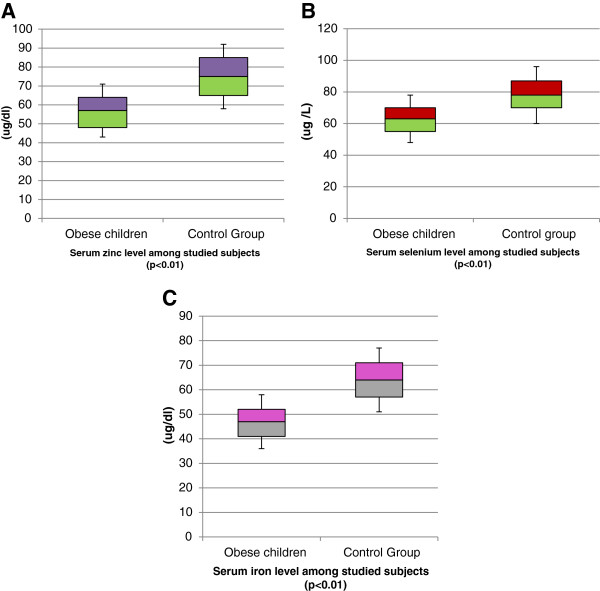
**Serum zinc, selenium, and iron level among studied subjects. A** Serum zinc level among studied subjects. **B** Serum selenium level among studied subjects. **C** Serum iron level among studied subjects.

The obese children had significantly lower median serum zinc, selenium and iron levels compared to non-obese controls (57 ± 14 vs 75 ± 17 ug/dl); (63.6 ± 15 vs 78.3 ± 18 ug/L); and (46.8 ± 11 vs 63.5 ± 13 ug/dl); respectively (*all P* < 0.01) (Figure 1A, B, C) respectively.

Simple correlation analyses were performed to investigate the association of serum trace elements levels with BMI and serum leptin level among studied subjects. The BMI was positively correlated with serum leptin level (*r* = 0.539*, P* < 0.01). Both serum Zn and iron levels showed significant negative correlations with BMI (*r* = −0.646*, P* < 0.01 and *r* = −0.479*, P* < 0.05; respectively) in the obese children. On the other hand, no significant correlations were found between serum Cu, Se or Cr levels and BMI in the obese children (all *P* > 0.05; Table 3).

**Table 3:** Correlations of serum trace elements levels with BMI and serum leptin in the obese children

	***Body mass index***		***Serum leptin***	
	***r***	***p***	***r***	***p***
*BMI (kg/m*^*2*^*)*	-	-	0.539	<0.01
*Serum leptin (ng/mL)*	0.539	<0.01	-	-
*Serum zinc (ug/dl)*	−0.646	<0.01	−0.746	<0.01
*Serum copper (ug/dl)*	0.112	>0.05	−0.245	>0.05
*Serum selenium (ug/l)*	−0.108	>0.05	−0.078	>0.05
*Serum iron (ug/dl)*	−0.479	<0.05	−0.245	>0.05
*Serum chromium (ng/ml)*	−0.063	>0.05	−0.078	>0.05

BMI, body mass index. Values are Correlation coefficient *r* (*Pearson’s Correlation* analysis). *P* value < 0.05 indicates a significant difference.

Serum leptin showed significant negative correlation with serum zinc levels in the obese children (*r* = −0.746; *P* < 0.01). On the other hand, no significant correlations were observed between serum leptin and serum Cu, Se, Fe or Cr levels in the obese children (all *P* > 0.05; Table 3).

On the contrary, no significant correlations were observed between BMI or serum leptin and studied trace elements in the non-obese controls (all *P* > 0.05).

To maximize the clarity in Tables 4 and 5 only statistically significant coefficients were included.

**Table 4:** Correlations of serum trace elements with lipid profile in the obese children and non-obese controls

	**Obese children (n=80)**				**Controls (n=80)**			
	**TGs**	**TC**	**LDL-C**	**HDL-C**	**TGs**	**TC**	**LDL-C**	**HDL-C**
**Serum zinc**	−0.268	−0.454*	−0.254	0.608**	−0.056	−0.115	−0.098	0.197
**Serum copper**	0.193	0.038	0.078	−0.153	0.137	0.069	0.037	−0.067
**Serum selenium**	−0.148	−0.197	−0.165	0.234	−0.091	−0.087	−0.165	0.184
**Serum iron**	0.094	0.158	0.186	−0.211	0.038	0.105	0.143	−0.178
**Serum chromium**	−0.135	−0.118	−0.156	0.209	−0.112	−0.087	−0.068	0.149

TGs, triglycerides; TC, total cholesterol; LDL-C, low density lipoprotein- cholesterol; HDL-C, High density lipoprotein- cholesterol. Values are Correlation coefficient *r* (*Pearson’s Correlation* analysis). *P < 0.05; **P < 0.01.

**Table 5:** Correlations of serum trace elements with glycaemia control parameters in obese children and non-obese controls

	**Obese children (n=80)**			**Controls (n=80)**		
	**Glucose**	**Insulin**	**HOMA-IR**	**Glucose**	**Insulin**	**HOMA-IR**
**Serum zinc**	−0.326*	−0.235	−0.469*	−0.079	−0.063	0.096
**Serum copper**	−0.105	−0.285	−0.163	−0.065	0.191	0.234
**Serum selenium**	0.203	0.119	0.635**	0.073	0.144	−0.208
**Serum iron**	−0.181	−0.154	−0.148	0.134	−0.235	−0.265
**Serum chromium**	0.143	−0.268	−0.123	−0.078	0.156	−0.087

HOMA-IR, homeostasis model assessment of insulin resistance.

Values are Correlation coefficient *r* (*Pearson’s Correlation* analysis)*.*

*P< 0.05; **P< 0.01.

In the obese children, serum Zn showed significant negative correlations with serum TC levels (*r* = −0.454, *P* < 0.05; Table 4). Otherwise, serum Zn levels showed significant positive correlation with HDL-C levels (*r* = 0.608*, P* < 0.01). By contrast, no significant correlation was found between serum Cu, Se, Fe or Cr levels and lipid profile in the obese children (all *P* > 0.05; Table 4).

Serum Zn levels were negatively correlated with fasting glucose levels and HOMA-IR values in the obese children (*r* = −0.326, *P* < 0.05 and *r* = −0.469, *P* < 0.05; respectively; Table 5). Of note, serum Se levels showed significant positive correlations with HOMA-IR values in the obese children (*r* = 0.635, *P* < 0.01). On the other hand, no significant correlation was found between serum Cu, Fe or Cr levels and glycaemia control parameters in the obese children (all *P* > 0.05; Table 5).

## Discussion

Recent evidence suggests that deficiencies of some micronutrients are related to obesity and fat deposition [15]. Few studies about the role of Zn in childhood obesity were published [16,17]. Some demonstrated a reduction in serum concentration of zinc in obese children [18]. Our study confirms the significantly lower serum Zn level in the obese children compared to the non-obese controls (57 ± 14 vs 75 ± 17 ug/dl; *P* < 0.01). Further, we observed a significant negative correlation between serum leptin and zinc levels in the obese children (*r* = −0.746; *P* < 0.01). Zinc, in particular, takes part in the metabolism of hormones involved in the pathophysiology of obesity. It has been observed that zinc concentration is directly associated with serum leptin concentration, an adipokine associated with satiety [19]. This association could be explained by the effect of zinc-α2-glycoprotein (ZAG) on leptin concentrations. ZAG is an adipokine involved in lipolysis in the adipocyte that is down-regulated in obesity. In obese individuals, low ZAG gene expression is associated with low serum adiponectin and high plasma leptin levels, and may play an important role in the pathogenesis of obesity [20]. Chen et al., suggested in their study that leptin resistance that occurred in obesity might have resulted from zinc deficiency [21]. On the contrary, no differences were observed in zinc concentrations between obese and non-obese Turkish children [22].

Cu is a component of antioxidant enzymes that act to protect the body against the action of free radicals, especially in cardiovascular diseases. An imbalance in the metabolism of Cu might trigger hypercholesterolemia and disorders in oxidative stress [23]. In trace element metabolism the best known interaction is the reported antagonism between zinc and copper [24]. The present study showed that serum Cu level in the obese children was significantly higher than those in healthy controls (*P* < 0.01). This agreed with Lima et al. who reported that copper concentrations in plasma in the overweight and obese groups were significantly higher than those in the control group [25]. The results found by the authors suggest that excess weight associated with lipid metabolism disorders might predispose to changes in Cu concentrations in plasma indicating a possible mechanism of this mineral, contributing to peroxidation or acting as an antioxidant. Clouet et al. [26] pointed out that, similar to iron, copper may also have an indirect impact on fatty acid oxidation and ATP production in mitochondria, helping the body’s metabolism to break down fatty acids and reduce fat.

Selenium, which is nutritionally essential for humans, is a constituent of more than two dozen selenoproteins (such as glutathione peroxidase GPx and selenoprotein P). These selenoproteins offer antioxidant protection against free radicals and other damaging reactive oxygen species [27] which are associated to the metabolic complications of obesity. In our study obese children had lower serum selenium levels compared to non-obese peers (63.6 ± 15 vs 78.3 ± 18 ug/L; *P* < 0.01). These results were concordant with a recent study by Ortega et al., who found that children with excess of weight have a poorer selenium status than children of normal weight, which can contribute to poor antioxidant protection [28]. By contrast, Bouglé et al., reported that none of their obese subjects did present with low Se levels; and their overall good Se status is supported by the normal activity of GPx [17].

In our study, significantly decreased serum Fe levels in obese children than those of the healthy controls (46.8 ± 11 vs 63.5 ± 13 ug/dl; *P* < 0.01); were in agreement with the findings of many other investigators [29,30]. Of note, there was no significant difference between obese children and non-obese peers as regards to serum ferritin levels (*P* > 0.05). Previous studies define Fe status by its serum levels [29,30] when considering ferritin level, the prevalence of iron deficiency is quite lower. It remains unclear, however, if the lower serum iron and elevated ferritin level seen in obesity are most reflective of a functional iron deficiency related to an inflammatory state, or if obesity is also a risk factor for true iron deficiency [31].

The significance of chromium as a trace nutrient is well documented and its function in the control of glucose and lipid metabolism has been claimed [32]. Our results indicated no association between serum chromium levels and obesity.

In our study, obese children have significantly higher TGs, TC and LDL-C, whereas HDL-C was significantly lower compared to non-obese peers (*P* < 0.01). In contrast, Woo et al., studied a cohort of obese subjects of Chinese ethnicity that did not show elevated cholesterol levels compared to a control group [33]. In addition, our results indicate that there are only few statistically significant correlations between trace elements and lipid profile was discovered in the obese children while there is no such correlation in healthy controls. In the obese children, we observed a significant negative correlation between serum Zn and TC levels (*r = −*0.454; *P* < 0.05) and a significant positive correlation existed between serum Zn and HDL-C levels (*r =* 0.608; *P* < 0.01); meanwhile the control group showed no such correlation. These findings indicate the possible effect of Zn level on serum lipid profile and this effect may be due to the role of Zn as an antioxidant. Thus, the decrease in Zn level in obese children may lead to increased lipid peroxidation and leading to increased levels of TC, TG and LDL-C. These results are concordant with those of a recent report by Gunasekara et al. who found that treatment with Zn reduced TC, TG, and LDL-C plasma levels and increased HDL-C levels [34].

Our data showed no significant correlation between serum Cu, Se, Fe, Cr and lipid profile parameters in the obese children (*P* > 0.05) which confirm the results of previous studies [17,22].

In our study, association of trace elements levels with glycaemia control parameters especially HOMA-IR values was assessed. Negative correlations were found between serum Zn levels and both fasting glucose and HOMA-IR in the obese children (*r* = −0.326, *P* < 0.05 and *r* = −0.469, *P* < 0.05; respectively). Zinc plays a major role in the stabilization of insulin hexamers and the storage of hormone in the pancreas [35] and its deficiency seems to impair release of insulin [8]. In the present study serum Se levels showed significant positive correlations with HOMA-IR values in the obese children (*r =* 0.635; *P* < 0.01). These results are concordant with those of a recent report by Stranges et al., who found a significant positive association between plasma Se and glucose levels at both baseline and follow up. They stated that high-Se diets may stimulate the release of glucagon, promoting hyperglycemia, or induce overexpression of glutathione peroxidase-1 and other antioxidant selenoproteins resulting in insulin resistance and obesity [36].

Our study pointed out that the consequences of changes in serum trace elements during childhood are much more important for the health of the public than is generally realized. Trace elements deficiencies may increase the risk of obesity and related diseases. So improving their status could give an opportunity to influence the clinical course of obesity [17]. There are some new studies in literature investigating the serum trace elements in obese adolescents and adults [37,38], However to the best of our knowledge, this was the first study of these trace elements in obese Egyptian children. The small sample size was one of our limitations in this study; we suggest that multicenter approaches may be necessary to attain larger sample size. Due to the lack of evaluations on metabolic risks and pubertal status; as all our subjects were in the pre pubertal stage, it was not possible to discuss more interesting and deeper findings.

The lack of longitudinal data showing whether weight loss can reverse the values of these trace elements; was another limitation in our study.

## Conclusion

The obese children may be at a greater risk of developing imbalance (mainly deficiency) of trace elements which may be playing an important role in the pathogenesis of obesity and related metabolic risk factors.

## Abbreviations

TE: Trace elements; ELISA: Enzyme-linked immunosorbent assay; TC: Total cholesterol; TGs: Triglycerides; BMI: Body mass index; HOMA-IR: Homeostasis model assessment of insulin resistance; HDL: High-density lipoprotein; LDL: Low-density lipoprotein.

## Competing interests

The authors declare that they have no competing interests.

## Authors’ contributions

*SFA* designed the study, collected samples, performed the statistical analysis, wrote discussion, and submitted the manuscript. *SS and WE* reviewed the results and discussion. *ME and LS* participated in the design of the study and helped to draft the manuscript*. A E* conceived of the study and coordinated the sample collection and analysis. All authors read and approved all the manuscript*.*
